# Psychometric Properties of the Healthy Lifestyle Questionnaire for Ecuadorian University Students (EVS-EUE)

**DOI:** 10.3390/ijerph18031087

**Published:** 2021-01-26

**Authors:** Mario Alvarez-Alvarez, Ricardo de la Vega-Marcos, Ruth Jiménez-Castuera, Marta Leyton-Román

**Affiliations:** 1 Research Group in Physical Activity and Sport Sciences, Universidad Politécnica Salesiana, Cuenca 010105, Ecuador; malvareza@ups.edu.ec; 2 Department of Physical Education, Sport and Human Movement, Autonomous University of Madrid, 28049 Madrid, Spain; ricardo.delavega@uam.es; 3 Didactic and Behavioral Analysis in Sport Research Group, Faculty of Sports Science, University of Extremadura, 10003 Cáceres, Spain; ruthji@unex.es; 4 Sport of Studies Center, Rey Juan Carlos University, 28933 Madrid, Spain

**Keywords:** eating habits, resting habits, instrument, university students, physical activity, psychometrics

## Abstract

University students are considered a key population in promoting and establishing healthy lifestyles that will ensure a full life for the next generations. The purpose of this study was to do a cultural and linguistic adaptation of the healthy lifestyle questionnaire for Ecuadorian university students (EVS-EUE). Two thousand, one hundred and eight (2108) students from 17 to 19 years old (27%), 20 to 24 years old (57%), and over 24 years old (16%) participated (*M* = 21.81 years; *SD* = 3.04). A confirmatory factor analysis, internal consistency analysis, and concurrent validity were conducted. The results of the EVS-EUE Questionnaire presented adequate values (χ^2^/d.f = 9.02, Comparative Fit Index (CFI) = 0.96, Incremental Fit Index (IFI) = 0.96, McDonald Fit Index (MFI) = 0.91, Adjusted Goodness of Fit Index (AGFI) = 0.94, Root Mean Square Error of Approximation (RMSEA) = 0.06, Standardized Root Mean Square Residual (SRMR) = 0.03). The internal consistency showed values above 0.70, and analyzed its concurrent validity, reaching adequate values. This study has provided a valid and reliable questionnaire to evaluate healthy lifestyles in the Ecuadorian population.

## 1. Introduction

Non-communicable diseases (NCDs), including different types of cancer [1], are one of the world’s major health problems and are involved in almost two-thirds of all deaths, accounting for 80% of the disease burden in low- and middle-income countries, such as Ecuador. The relationship of these diseases to unhealthy lifestyles, like smoking, alcohol consumption, unhealthy diet, and physical inactivity [2], which are the four main behavioral risk factors for this group of diseases, has been extensively studied [3].

The scientific evidence on the prevalence of unhealthy lifestyles in university students, in the international area, is alarming, with percentages ranging from 7% to 44.1% for smoking [4,5,6,7], 50.1% to 69.3% for alcohol consumption [4,5,7,8], 44.1% to 92% for low fruit and vegetable consumption [5,7,9], 31.8% to 57.8% for physical inactivity [4,7], poor sleep quality from 26% to 61.9% [4,7,9,10,11], and 14.5% to 33.8% for unhealthy eating habits [9,12]; the most worrying aspect of this situation is the fact that these lifestyles are directly related to higher mortality [3,13,14]. For these reasons, scientific evidence shows that they are a serious public health problem that could contribute to the increase of multifactorial diseases in the population [15], even more so in this time of pandemic caused by COVID-19, which has become a barrier to compliance with healthy guidelines and has decreased healthy lifestyles [16] and therefore increased risk factors for cardiovascular disease [17].

Understanding the changes in lifestyles that occur in the different periods that an individual goes through is transcendental, since it provides valuable information for the investigation of healthy behavior. It also allows a better development of intervention programs in the field of public health that can influence, at the preventive level, chronic multifactorial diseases and their effects [18]. In addition, it allows to mitigate, through interventions, the negative consequences on lifestyles resulting from confinement by communicable diseases such as COVID-19 [19].

Childhood and adolescence are considered the foundation for healthy lifestyle choices and behaviors in adulthood [20]. However, the emerging adulthood that comprises from 18 to 30 years old [21] is considered a culturally constructed evolutionary period that coincides with the university years [22], and that presents five general characteristics: (a) exploration of identity; (b) instability; (c) great optimism and possibilities; (d) being self-centered; and (e) a stage to feel in the middle, between adolescence and maturity. Additionally, it is considered not only an era of possibilities and freedoms, but also of behavioral risk factors [23]. Therefore, this stage is the ideal time to implement strategies to consolidate healthy lifestyles [24], even more so because this population is considered a social care group [2] and is affected by different negative alterations such as a decrease in physical activity [25], mood disorders, anxiety, and unhealthy behaviors, which could be avoided through an analysis of their lifestyles and early interventions [26], thus setting a path for healthy aging [27].

In this sense, several studies have shown a direct relationship between physical activity and the components of healthy lifestyles, with one becoming the way to reach the other [28,29]. The lower the levels of physical activity, the poorer the healthy lifestyles [30] and the poorer the adherence to them [31]. In fact, healthy lifestyles consider physical activity a fundamental part of themselves [29].

So far in Ecuador, no instruments have been validated to assess healthy lifestyles in university students. However, several instruments have been created and validated in other countries to study this issue. Among them, there is the “Lifestyle Questionnaire for Young University Students (CEVJU-R)” by its Spanish acronym, in which 1485 students participated in a first phase, and in a later phase, 1811 university students from four private and public institutions in Colombia. This instrument consists of 156 items that measure the dimensions of sexuality, perceived emotional state, consumption of alcohol, tobacco and illegal drugs, coping, sleep quality, interpersonal skills, physical activity, leisure time, eating disorders, self-care, and diet [32]. The “Health-Promoting Lifestyle Profile II”, which was validated for Spanish university students and included a sample of 1219 participants, has also been in widespread use. It assesses the dimensions of self-realization, responsibility for health, exercise, nutrition, interpersonal support and stress management [33]. This same questionnaire was validated for the university population of Portugal, with a sample of 12,700 participants [34], and for the population of Malaysia, with 997 university students [35].

The “Healthy Lifestyles Scale for University Students (HLSUS)” was validated with 5523 university students in China and focused on evaluating eight dimensions: social support, life appreciation, regular behavior, nutritional behavior, physical exercise, health risk behaviors, stress management, and responsibility for health [36]. Later, it was translated and validated into Spanish with 530 women aged between 18 and 25 years, from six academic areas of two Mexican public universities [37].

There is also the Portuguese version of the “Fantastic Lifestyles” questionnaire, which was validated with 707 university students in the area of health in Portugal, and measures comportments related to family and friends, physical activity/association, nutrition, consumption of tobacco, alcohol and other drugs, sleep, stress, work, personality, and health-oriented and sexual behaviors among others [38].

In relation to the healthy lifestyle questionnaires for other age ranges, the Portuguese version of the “Healthy Lifestyles Questionnaire” (HLQ), which measures a balanced diet, respect for mealtimes, tobacco consumption, alcohol consumption, consumption of other drugs, and rest habits, was validated with a sample of 348 veteran athletes aged between 30 and 60 years [39]. This same questionnaire was authenticated in the Spanish population [40], with a sample of 14- to 88-year-olds.

The objective of this study is to validate this last questionnaire with the Ecuadorian university population, analyzing its concurrent validity by means of a correlation analysis with the different levels of physical activity, contributing to the adaptation of this instrument to the Ecuadorian scientific community for its use in the context of people’s health and integral wellbeing.

## 2. Materials and Methods

### 2.1. Design

This is an instrumental study [41] aimed at analyzing the psychometric properties of an evaluation instrument that was applied to students from four universities located in the Andean region, southern mountain range of Ecuador, specifically in the city of Cuenca.

### 2.2. Participants

From a reference population of 38,842 students from the participating Higher Education Institutions, a sample of 2264 participants (95% confidence level, 2% margin of error, and 50% expected frequency) was determined using EPI INFO version 7.2 Software for Windows. Finally, those who answered the questionnaires were 2108 students (*M* = 21.81 years; *SD* = 3.04) with age ranges from 17 to 19 years (27%), 20 to 24 years (57%), and over 24 years (16%), of which 990 (46.86%) were male, 1125 (53%) female, and three (0.14%) were considered to be of another gender. The eligibility criteria used were: (1) full-time undergraduate students, (2) aged between 17 and 30, and (3) of Ecuadorian nationality. With regard to the exclusion criteria: (1) undergraduate students in blended or virtual mode, (2) aged over 30, and (3) of a nationality other than Ecuadorian. The components of this sample group were selected by means of intentional, non-probability sampling by clusters, which was conducted equally by year of study and by university, in such a way that there was representativeness in the sample size [42].

### 2.3. Instruments

The entries were extracted from the Healthy Lifestyle Questionnaire (HLQ) in a Spanish population [40]. The instrument is called “Healthy Lifestyles Questionnaire in Ecuadorian University Students (EVS-EUE)”, and it is composed of 12 items. Five of them assess each of the factors related to a healthy lifestyle: tobacco consumption (e.g., “I smoke regularly”), rest habits (e.g., “ I sleep between 7 and 8 h at least five times a week”); regarding respect for meal times (e.g., “I eat breakfast, lunch, and snacks at the same time, at least five times a week”), and maintaining a balanced diet (e.g., “I eat five portions of fruit and vegetables each day at least five times a week”). The answers were collected on a Likert scale, with a range of scores from 1 (strongly disagree) to 5 (strongly agree).

To determine the concurrent validity, the Level of Physical Activity was measured, and the International Physical Activity Questionnaire (IPAQ), a self-administered short format of the last seven days, was used [43]. This self-report is composed of seven elements that assess the physical activity performed by the subject during a minimum of 10 min, in four different domains (transport, occupation, house/lawn, and leisure time) during the last seven days. The frequency and duration of vigorous activity performed (8.0 Metabolic Equivalent of Task (METs)/minutes/week), moderate activity (4.0 METs/minutes/week), and low intensity walking (3.3 METs/minutes/week) are assessed.

### 2.4. Procedure

The study was approved by the Research Ethics Committee of the Universidad Autónoma de Madrid under the registration number CEI-103-1980, following the guidelines of the Declaration of Helsinki. All participants were treated according to the ethical guidelines of the American Psychological Association regarding participant consent, confidentiality, and anonymity. Written informed consent was obtained from all participants.

For the linguistic and cultural adaptation, seven expert judges evaluated the questionnaire: a nutritionist, two psychologists, a linguist, and three physical activity and sport specialists. The degree of item-construct adjustment, the syntactic and semantic suitability, and the typical expressions of the Ecuadorian socio-cultural context were evaluated. Cognitive interviews were also conducted with eight students from the target population (four men and four women), since a review of the results of this phase against the original version of the instrument is key to ensuring cultural relevance [44]. Once the analysis of the results of the cognitive interviews was done, the application of the pilot test was performed with university students of different ages (target population) in order to evaluate the understanding of the slogan, the elements included in the instrument, and the duration of the application of the instrument. This test indicated that the items could be understood by the participants of different ages, so the questionnaire could be applied without problems to all participants. At this stage, the two voices that participated in the adaptation (expert judges and the target population) were integrated and constituted independent sources to guarantee the conceptual, cultural, and linguistic adequacy of the questionnaire in our context [45].

For the application phase to all participants, previous contact with the directors of the different centers was made in order to ask for their collaboration in the study. The application of the instruments (printed format) was carried out in the presence of the head researcher to briefly explain the objectives and structure, as well as how to fill them in. During the completion process, the head researcher solved some problems and answered some questions that emerged. The place of application was the classroom, with prior authorization from the teacher on duty. The time for self-fulfillment was approximately ten minutes.

### 2.5. Data Analysis

Each variable passed the tests of normality through the Kolgomorov–Smirnov test and homogeneity of variances through the Levene test [46], which led to the use of parametric statistics.

Confirmatory factor analysis (CFA) was performed, taking into account a combination of indices [47]. The indicators recommended by Byrne [48] were followed to determine the fit of the scale: χ^2^, χ^2^/d.f., CFI (Comparative Fit Index), IFI (Incremental Fit Index), MFI (McDonald Fit Index), SRMR (Standardized Root Mean Square Residual), and AGFI (Adjusted Goodness of Fit Index). Jöreskog et al. [49] recommend that the χ^2^/d.f., present values below 2, which indicates a very good fit of the model, while values below 5 are considered acceptable [50]. In the incremental indices (CFI, IFI, AGFI), values above 0.90 are considered acceptable, but if they are above 0.95, they are considered good [50,51]. RMSEA and SRMR error rates should be less than 0.08 [52,53].

Subsequently, a descriptive and internal consistency analysis was carried out. In the descriptive analyses, asymmetry and kurtosis were studied. Two indices were used for the reliability analysis, Cronbach’s Alpha (α) (equal to or higher than 0.70) [54] and the Omega Coefficient (ω) [55], which also serves to check the internal consistency of the variables used in the research and, according to some authors [56], has shown evidence of greater accuracy. Additionally, in the McDonald Omega Coefficient, the established range is between 0 and 1, with the higher values, which give us more reliable measurements [56]. Finally, to determine the concurrent validity, an analysis of bivariate correlations of Pearson between the variables of the EVS-EUE Questionnaire and the levels of physical activity obtained through the IPAQ-short form was used.

Descriptive analysis, internal consistency, and concurrent validity were performed using the SPSS statistical package, version 21.0 for Windows (IBM, Armonk, NY, USA). To confirm the structure of the factors with their corresponding items, the CFA, the EQS software, version 6.1 for Windows (Multivariate Software, Inc., Los Angeles, IL, USA) was used.

## 3. Results

### 3.1. Confirmatory Factor Analysis

A CFA was carried out to evaluate the model of the EVS-EUE questionnaire. The standardized factor loads were all statistically significant (*p* < 0.01), so it can be concluded that the model at the analytical level presents satisfactory results (Table 1).

The global results of the model indicated an optimal fit: χ^2^/gl = 9.02, IFC = 0.96, IFI = 0.96, MFI = 0.91, AGFI = 0.94, RMSEA = 0.06, and SRMR = 0.03. With these results, it can be concluded that the structural model has a satisfactory overall fit.

### 3.2. Descriptive and Internal Consistency Analysis

As can be seen in Table 2, in relation to Cronbach’s Alpha reliability analysis, the balanced diet factor showed a lower reliability than recommended (0.70) [54], but given the small number of items that make up the factor (three), the internal consistency observed can be marginally accepted [57,58]. In relation to the McDonald Omega Coefficient analysis, all the indices are within the established range (between 0 and 1) [56]. According to the rules of normality [59], all the variables comply with the univariate normality, since the values of asymmetry were below 2, and those of kurtosis, below 7.

### 3.3. Current Validity

The quantification of the concurrent validity was evaluated by observing the correlation between constructs. The data obtained from Pearson’s correlation analysis revealed that with respect to the variables of the IPAQ-short form questionnaire, significant and positive correlations were found between a balanced diet, respect for mealtimes, and rest habits and levels of mild, moderate, and vigorous physical activity, as well as total physical activity, although at a very low correlation [60] (Table 3).

## 4. Discussion

This study analyzed the validity of the Healthy Lifestyles Questionnaire in Ecuadorian University Students (EVS-EUE), based on the Healthy Lifestyles Questionnaire by Leyton et al. [40], with the factors balanced diet, respect for mealtimes, tobacco consumption, and rest habits. The psychometric properties of the EVS-EUE Questionnaire have been examined through the CFA, reliability, and concurrent validity analysis. The results of the CFA revealed that the structure was adequate and the adjustment rates were acceptable [53]. In relation to the reliability analyzed through McDonald’s Omega Coefficient and Cronbach’s Alpha Coefficient, high levels of internal consistency were shown. Regarding concurrent validity, the data obtained from Pearson’s correlation analysis revealed that with respect to the variables of the IPAQ-short form questionnaire, it was corroborated that there are significant and positive correlations between a balanced diet, respect for mealtimes, and rest habits with the levels of mild, moderate, and vigorous physical activity, as well as with total physical activity; however, as indicated above, the correlation was very low.

These results are higher than those obtained by the Young University Lifestyle Questionnaire (CEVJU-R) validated with Colombian students [32]; nevertheless, regarding balanced diet, the coefficient is below the value to be considered acceptable (<0.70), which differs from the original version [40], but is similar to the value of the Health-Promoting Lifestyle Profile II (HPLPII) Spanish version regarding nutrition [33].

The results of correlations found in the validation of this questionnaire corroborate the relationship between the acquisition of healthy eating habits and physical activity, highlighting a directly proportional relationship between these two variables that would be beneficial for healthy lifestyles as a whole [29,30]. With respect to this relationship, according to Jezewska-Zychowicz et al. [61], healthy eating habits are more likely to coexist with moderate and high physical activity in the context of work and school, thus allowing them to be used in interventions that seek to prevent the risks or mitigate the negative effects of chronic multifactorial diseases [19].

Another important aspect that strengthens existing scientific evidence is that rest habits have a significant relationship with physical activity levels. These results are similar to those reported by Lin et al. [62], who indicated that sleep duration is significantly associated with levels of light and moderate to vigorous physical activity, and conversely, poor sleep quality is associated with insufficient physical activity [63], which is very important, as it has been shown to be directly related to weight gain, obesity, cardio-metabolic disease, mortality, and other negative health outcomes [64,65].

As far as tobacco consumption and physical activity are concerned, in this study, no significant relationships were found. This disagrees with the results of Carballo-Fazanes et al. [66], who found that smoker students present lower levels of physical activity, that is, there is an inverse association [67], and that smoking, among other aspects, is a suboptimal predictor of physical activity [4]; however, there are studies that indicate that this relationship is not always significant in this group, since it may be affected by other variable, such as the level of education and the performance of national prevention programs in these populations during the studies [68].

Therefore, a balanced diet, respect for mealtimes, adequate rest habits, and the non-use of tobacco by university students could make it possible to achieve adequate levels of physical activity for proper health. This is positive, since low levels of physical activity are associated with increased mortality from noncommunicable diseases [1,2].

Regarding the general description of the healthy lifestyles of Ecuadorian university students, the highest values are in respect of mealtimes and rest habits. However, these scores are lower than those obtained by Batista et al. [39] with veteran athletes from Portugal and those of Leyton et al. [40] with a general Spanish population. These results would further support findings that indicate a reduction in healthy lifestyles in emerging adulthood [25,26,69] and therefore an increase in risk factors that will be a key aspect of public health for generations to come [27].

Among the limitations of this study, we can mention that, despite having been applied in all the universities of the third largest city in Ecuador, it requires extending the sample to universities in the coastal region. Another limitation has to do directly with the questionnaire, as it does not address all the dimensions considered within healthy lifestyles, such as the consumption of alcohol and other drugs, which is very common among Ecuadorian university students, and which should be included in the future within this instrument. However, it measures two of the fundamental aspects of healthy lifestyles, which are often used to prevent or mitigate behaviors associated with chronic non-communicable diseases [31].

## 5. Conclusions

In conclusion, this study has provided a valid and reliable questionnaire to assess the healthy lifestyles of Ecuadorian university students between the ages of 17 and 30. In spite of the mentioned limitations, the EVS-EUE questionnaire can even become a very important tool for evaluation, being the only instrument of this nature that has been validated in Ecuador so far. It also establishes the beginning of future adaptations for other population groups in this context. These results also have important implications in the educational field, since they will allow universities to implement intervention strategies that focus not only on physical activity, but also on other lifestyles. In addition, it serves as an instrument to make the different initial diagnoses and controls in the interventions that are made in this population group, because it analyzes variables directly related to health.

## Figures and Tables

**Table 1:** Standardized Factorial Loads of the HLQ-EUS Questionnaire (*n* = 2108).

Factor	Item	CF
Balanced Diet	5	0.43 *
	8	0.67 *
	12	0.52 *
Respect for Mealtimes	4	0.67 *
	6	0.67 *
	11	0.79 *
Tobacco consumption	1	0.31 *
	7	0.36 *
	10	0.33 *
Rest Habits	2	0.65 *
	3	0.66 *
	9	0.61 *

Note: CF, Standardized Factorial Load * *p* < 0.05.

**Table 2:** Descriptive statistics, asymmetry, kurtosis, and reliability analysis of the variables of the HLQ-EUS Questionnaire (*n* = 2108).

Factors	M	SD	Asymmetry	Kurtosis	*α*	*ω*
Balanced Diet	2.67	0.90	0.24	−0.32	0.64	0.81
Respect for Mealtimes	2.96	1.13	0.06	−0.96	0.77	0.81
Tobacco consumption	1.65	0.97	1.56	1.71	0.85	0.91
Rest Habits	2.71	1.05	0.34	−0.59	0.85	0.87

Note. M = Mean; TD = Typical Deviation; α = Cronbach’s Alpha; ω = Omega Index.

**Table 3:** Descriptive statistics and correlation analysis between variables.

		Range	M	SD	1	2	3	4	5	6	7	8
1.	Balanced Diet	1–5	2.67	0.90	-	0.651 **	0.076 **	0.470 **	0.019	0.110 **	0.115 **	0.080 **
2.	Respect for Mealtimes	1–5	2.96	1.13	-	-	0.023	0.476 **	−0.006	0.061 **	0.083 **	0.049 *
3.	Tobacco Consumption	1–5	1.65	0.97	-	-	-	0.056 *	0.021	−0.019	−0.041	−0.014
4.	Rest Habits	1–5	2.71	1.05	-	-	-	-	−0.011	0.051 *	0.73 **	0.036
5.	Mild Physical Activity (MET-min/week.)	1–5	1.24	0.67	-	-	-	-	-	0.184 **	0.145 **	0.681 **
6.	Moderate Physical Activity (MET-min/week.)	1–5	1.07	0.36	-	-	-	-	-	-	0.266 **	0.579 **
7.	Vigorous Physical Activity (MET-min/week.)	1–5	1.16	0.49	-	-	-	-	-	-	-	0.500 **
8.	Total Physical Activity (MET-min/week)	1–5	1.09	0.33	-	-	-	-	-	-	-	-

Note: * *p* < 0.05; ** *p*< 0.01.

## Data Availability

The data presented in this study are available on request from the corresponding author. The data are not publicly available for privacy reasons.
